# Chemical Composition, Antioxidant Activity and α-Glucosidase Inhibitory Activity of Chaenomeles Speciosa from Four Production Areas in China

**DOI:** 10.3390/molecules23102518

**Published:** 2018-10-01

**Authors:** Xuan Zheng, Hongwei Wang, Peng Zhang, Lin Gao, Ning Yan, Panpan Li, Xinmin Liu, Yongmei Du, Guoming Shen

**Affiliations:** 1 Tobacco Research Institute of Chinese Academy of Agricultural Sciences, Qingdao 266101, Shandong, China; zhengxuan1201@163.com (X.Z.); whw20051256@163.com (H.W.); zhangpeng@cass.cn (P.Z.); gaolin@caas.cn (L.G.); yanning@caas.cn (N.Y.); 13165154365@163.com (P.L.); liuxinmin@caas.cn (X.L.); 2 Graduate School of Chinese Academy of Agricultural Sciences, Beijing 100081, China

**Keywords:** *Chaenomeles speciosa*, chemical composition, antioxidant activity, *α*-glucosidase inhibitory activity

## Abstract

*Chaenomeles speciosa* (Sweet) Nakai is a medicinal plant. Until date, there are no studies focusing on comparing the chemical profiles, antioxidant activity and *α*-glucosidase inhibitory activity of the dried fruits of *C. speciosa* from different production regions. In the study, we investigated the chemical components of dried fruits of *C. speciosa* from Yunnan, Chongqing, Zhejiang and Anhui provinces in China in relation to the antioxidant activity and *α*-glucosidase inhibitory activity. *C. speciosa* from Yunnan had higher total flavonoid (47.92 ± 3.79 mg/g), total polyphenol (29.15 ± 0.29 mg/g) and polysaccharide (27.60 ± 1.56 mg/g) contents than plants from other production areas. Samples from Yunnan, Zhejiang and Anhui (all > 3200 mg/kg) had higher free amino acid contents than those from Chongqing (2286.66 mg/kg). Oleanolic acid and ursolic acid levels were highest in samples from Zhejiang (555.98 ± 20.88 μg/g) and Anhui (321.06 ± 14.64 μg/g), respectively. *C. speciosa* from Chongqing had low total flavonoid, total polyphenol, polysaccharide, free amino acid, oleanolic acid and ursolic acid contents but high levels of palmitic acid (12.04 ± 0.02 mg/g) and stearic acid (2.23 ± 0.08 mg/g). Among four production areas, Yunnan represented the highest antioxidant activity and *α*-glucosidase inhibitory activity. In addition, correlation analysis revealed that total flavonoid, total polyphenol, polysaccharide and ursolic acid were the major components responsible for the antioxidant activity of *C. speciosa*, while total flavonoid and polysaccharide were the main contributors for *α*-glucosidase inhibitory activity of the plant. These results would be helpful for evaluating the quality of *C. speciosa* in the different production areas.

## 1. Introduction

The genus *Chaenomeles,* belonging to the Rosaceae family, is mainly distributed in Korea, Japan and China. There are five wild *Chaenomeles* species in China including *C. speciosa* (Sweet) Nakai, *C. thibetica* Yü, *C. cathayensis* (Hemsl.) Schneider, *C. sinensis* (Thouin) Koehne and *C. japonica* (Thunb.) Lindl. ex Spach*. C. speciosa* has traditionally been cultivated in Yunnan, Chongqing, Zhejiang and Anhui provinces and its dried fruits are commonly used in traditional medicine for the treatment of the common cold, asthma, hepatitis and rheumatoid arthritis [1]. The fruits of *C. speciosa* have reported anti-inflammatory, antinociceptive, antimicrobial, antioxidant, immunoregulatory, antitumor, hepatoprotective and antiparkinsonian activities [2,3,4,5]. 

Flavonoids, polyphenols, polysaccharides, free amino acids, organic acids and triterpenic acids have been isolated from the fruits of *C. speciosa*. The pharmacological properties of *C. speciosa* can be understood by identifying the chemical constituents of the plant. For example, phenolic compounds such as anthocyanins and flavonoids have shown anti-inflammatory, antitumoral, antimicrobial and antioxidant effects [6]. The phenolic hydroxyl groups in polyphenol compounds could lose electrons easily [7], which accounts for their antioxidant activity. Polysaccharides have exhibited antitumor, antioxidant and immunomodulatory properties [8,9]. Oleanolic acid (OA) and ursolic acid (UA) exhibit anti-inflammatory [10], antitumor [11,12] and *α*-glucosidase inhibitory effects [13,14]. Among these biological activities, antioxidant activity and *α*-glucosidase inhibitory activity of *C. speciosa* have attracted more and more attention from researchers. 

Ferric reducing/antioxidant power (FRAP), 2,2-diphenyl-1-picrylhy-drazyl (DPPH), 2,2’-azino-bis(3-ethylbenzothiazoline-6-sulfonic acid) (ABTS), super oxygen anion (SOA) and hydroxyl free radical assays were the commonly used approaches for evaluating the antioxidant activity of *C. speciosa*. 4’-Nitrophenyl-beta-d-glucopyranoside (PNPG) is usually adopted for exploring the *α*-glucosidase inhibitory activity of the plant because it could be decomposed by *α*-glucosidase into 4-nitrophenol and the product could be further detected by microplate reader at 405 nm [14]. Recently, some investigations have been performed on the antioxidant activity and *α*-glucosidase inhibitory activity of *C. speciosa.* For examples, Xie et al. [8] isolated and characterized polysaccharides from the dried fruits of *C. speciosa*. The purified polysaccharides exhibited remarkable antioxidant activity to scavenge the DPPH, ABTS, SOA and hydroxyl radicals. Gao et al. [15] presented the effect of the solvents including water, different methanol aqueous and different ethanol aqueous on the chemical composition, antioxidant activity and *α*-glucosidase inhibitory activity of *C. speciosa.* The results revealed that 60% ethanol was the best choice for industrial production of *C. speciosa* extract with good antioxidant activity and *α*-glucosidase inhibitory activity. In addition, the same group [16] reported that the peels of *C. speciosa* performed better FRAP, DPPH and *α*-glucosidase inhibitory activities than the fleshes. 

Until date, there are no studies focusing on comparing chemical profiles, antioxidant activity and *α*-glucosidase inhibitory activity of the dried fruits of *C. speciosa* from different production regions. To address this issue, in the present study, we compared the contents of total flavonoid (TF), total polyphenol (TP), polysaccharide, free amino acid, organic acid, OA and UA as well as antioxidant activity and *α*-glucosidase inhibitory activity of *C. speciosa* from Yunnan, Chongqing, Zhejiang and Anhui provinces and examined the correlation of these chemical components with the antioxidant activity and *α*-glucosidase inhibitory activity of the plant. 

## 2. Results and Discussion

### 2.1. Chemical Composition of C. speciosa from Four Production Areas in China

#### 2.1.1. TF, TP and Polysaccharide Contents

**Figure 1:**
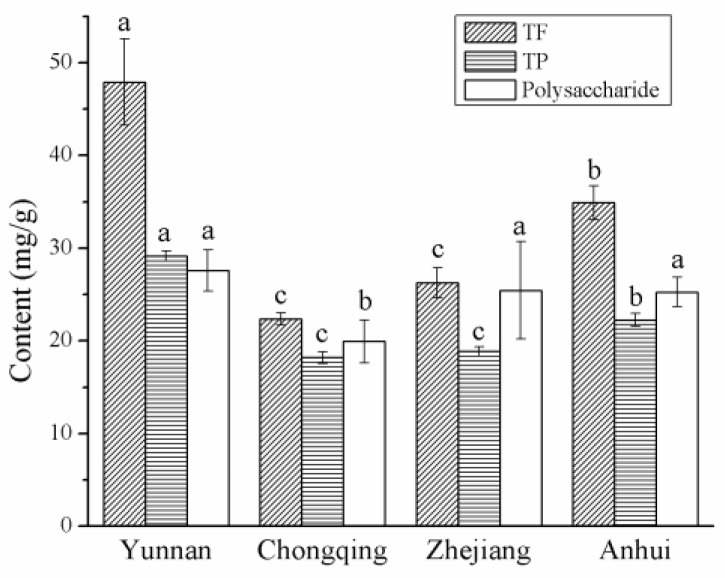
TF, TP and polysaccharide contents of the dried fruits of *C. speciosa* from four production areas in China. Results are shown as mean ± SD (*n* = 3). Different letters above each bar indicate significant differences (*p* < 0.05). TF, total flavonoid; TP, total polyphenol.

The dried fruits of *C. speciosa* from Yunnan had the highest TF content (47.92 ± 3.79 mg/g), which was about 2-fold higher than the level in plants from Chongqing (22.36 ± 0.53 mg/g) (Figure 1). TF contents of plants from Zhejiang (26.27 ± 1.34 mg/g) and Chongqing were similar and were lower than that of plants from Anhui (34.89 ± 1.49 mg/g). The dried fruits of *C. speciosa* from Yunnan had a higher TP content (29.15 ± 0.29 mg/g) than samples from Chongqing (18.19 ± 0.37 mg/g), Zhejiang (18.85 ± 0.24 mg/g) and Anhui (22.27 ± 0.49 mg/g). The TP contents from four production areas were significantly higher than the results (13.58 ± 0.13 mg/g) reported by Li et al. [17]. Gao et al. [13] investigated the effect of the drying methods, including freeze-drying and hot air drying, on the TP content of *C. speciosa.* It is found that all the fruits of *C. speciosa* processed by both the drying methods have lower TP content (ranging from 2.14 ± 0.10 to 3.28 ± 0.47 mg/g) than the samples from Chongqing in the work. The dried fruits of *C. speciosa* from Yunnan (27.60 ± 1.56 mg/g), Zhejiang (24.45 ± 3.70 mg/g) and Anhui (25.26 ± 1.31 mg/g) had similar polysaccharide contents, which were higher than that of plants from Chongqing (19.95 ± 1.88 mg/g). Thus, dried fruits of *C. speciosa* in Yunnan had the highest TF, TP and polysaccharide levels in four production areas.

#### 2.1.2. Free Amino Acid Content

The total amino acid content in samples from Chongqing (2286.66 mg/kg) was lower than that of samples from Yunnan (3411.60 mg/kg), Anhui (3349.83 mg/kg) and Zhejiang (3226.30 mg/kg) (Table 1). On the contrary, total essential amino acid content was higher in plants from Chongqing (823.60 mg/kg) than in those from Yunnan (632.91 mg/kg), Zhejiang (602.13 mg/kg) and Anhui (647.96 mg/kg). However, the abundance of specific essential amino acids did not reflect the total amino acid and total essential amino acid contents. That is, threonine (26.58 ± 0.88 mg/kg), valine (51.42 ± 1.97 mg/kg) and phenylalanine (107.75 ± 6.63 mg/kg) levels were higher in *C. speciosa* from Yunnan than in plants from the other three provinces, whereas the opposite trend was observed for methionine (30.29 ± 4.54 mg/kg), isoleucine (13.78 ± 0.75 mg/kg) and leucine (9.41 ± 1.49 mg/kg). The highest levels of isoleucine (30.43 ± 3.43 mg/kg) were found in samples from Chongqing, whereas leucine (34.23 ± 1.40 mg/kg) and lysine (44.07 ± 2.65 mg/kg) levels were highest in samples from Zhejiang. Methionine content (35.79 ± 0.62 mg/kg) was highest in plants from Anhui. Samples from Chongqing had the lowest threonine (5.94 ± 0.56 mg/kg) and phenylalanine (43.60 ± 2.46 mg/kg) contents, whereas valine (30.40 ± 0.62 mg/kg) and lysine (21.03 ± 1.74 mg/kg) levels were lowest in Zhejiang and Anhui samples, respectively.

#### 2.1.3. Organic Acid Content

We identified 19 organic acids in *C. speciosa* samples from four production areas, which is consistent with results reported in the literature [18] (Table 2). Malic acid constituted the largest fraction of total organic acids in samples from Yunnan (66.77%), Zhejiang (40.58%) and Anhui (63.06%). Oleic acid content represented about 41.77% of total organic acids in samples from Chongqing. Malic, citric, palmitic, linoleic, oleic and 10-hydroxy-hexadecanoic acids together accounted for over 90% of total organic acids in all samples from four production areas and were therefore the focus of quantitative analysis.

**Figure 2:**
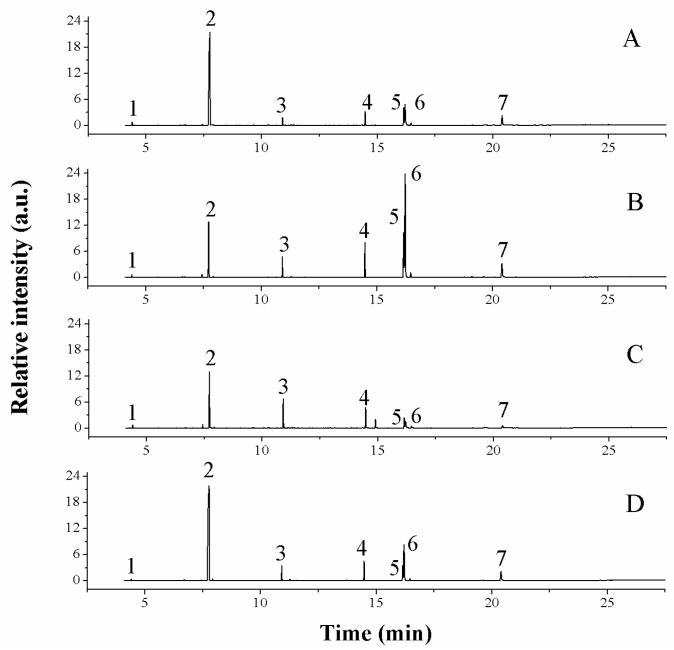
Chromatograms showing organic acid levels in the dried fruits of *C. speciosa* from four production areas. The production areas were as follows: (**A**) Yunnan; (**B**) Chongqing; (**C**) Zhejiang; (**D**) Anhui. The following organic acids were detected: 1, oxalic acid; 2, malic acid; 3, citric acid; 4, palmitic acid; 5, linoleic acid; 6, oleic acid; 7, octadecanoic acid.

Representative chromatograms of organic acids in *C. speciosa* samples are shown in Figure 2. Oxalic, malic, citric, palmitic, linoleic, oleic and stearic acids were identified by matching the retention times to those of known standards. Linoleic and oleic acids could not be quantified due to their co-elution. Therefore, we compared the contents of the remaining five baseline-separated organic acids in samples from four production areas (Table 3). *C. speciosa* from Yunnan (3.20 ± 0.05 mg/g) and Anhui (1.33 ± 0.08 mg/g) had the highest and lowest levels of oxalic acid, respectively. Malic acid levels in samples from Yunnan (124.96 ± 7.43 mg/g) and Anhui (139.91 ± 4.24 mg/g) were three times higher than those in plants from Chongqing (34.81 ± 0.35 mg/g) and Zhejiang (32.86 ± 0.05 mg/g). Citric acid content in plants from four production areas varied from 10.26–30.91 mg/g. *C. speciosa* from Chongqing had a higher palmitic acid content (12.04 ± 0.02 mg/g) than samples from Zhejiang (7.41 ± 0.07 mg/g), Anhui (7.45 ± 0.13 mg/g) and Yunnan (5.78 ± 0.28 mg/g). Stearic acid concentration was about two times higher in plants from Chongqing (2.23 ± 0.08 mg/g) than in those from Yunnan (1.05 ± 0.04 mg/g), Zhejiang (1.16 ± 0.10 mg/g) and Anhui (1.09 ± 0.01 mg/g). 

#### 2.1.4. OA and UA Contents

OA and UA were the important ingredients in *C. speciosa.* The dried fruits of *C. speciosa* from Zhejiang had the highest concentration of total triterpenic acids (720.89 μg/g), which was over twice that in samples from Chongqing (331.51 μg/g) (Table 4). Plants from Zhejiang and Yunnan had the highest OA (555.98 ± 20.88 μg/g) and UA (339.97 ± 11.86 μg/g) levels, respectively. In general, the concentration of UA was higher than that of OA in four production areas [1]. Gao et al. [13] also investigated the effect of the drying methods on OA and UA contents of *C. speciosa.* The OA and UA contents for all the dried fruits of *C. speciosa* ranged from 2.37 ± 0.01 to 17.38 ± 0.03 mg/g and 15.16 ± 0.10 to 2.52±0.03 mg/g, respectively. These results were at least four times more than the findings of the present study.

### 2.2. Antioxidant Activity and α-Glucosidase Inhibitory Activity of C. speciosa

**Figure 3:**
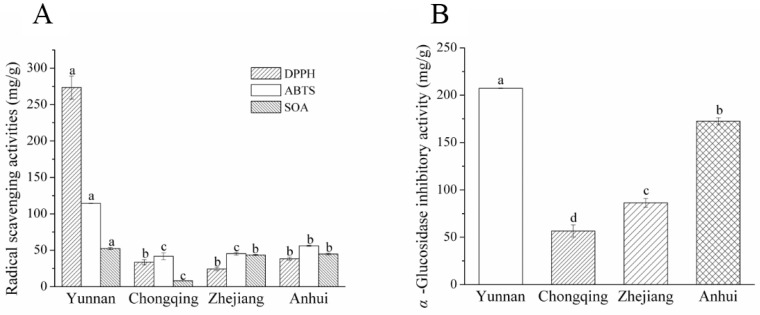
(**A**) DPPH, ABTS and SOA radical-scavenging activities and (**B**) α-glucosidase inhibitory activity of the dried fruits of *C. speciosa*. Results are shown as mean ± SD (*n* = 3). Different letters above each bar indicate significant differences (*p* < 0.05). DPPH, 2,2-diphenyl-1-picrylhy-drazyl; ABTS, 2,2’-azino-bis(3-ethylbenzothiazoline-6-sulfonic acid); SOA, super oxygen anion.

DPPH, ABTS and SOA radical-scavenging assays were adopted for evaluating the antioxidant capacities of the dried fruits of *C. speciosa* (Figure 3A) from four production areas. The results showed that *C. speciosa* from Yunnan had the highest DPPH (273.30 ± 15.59 mg/g), ABTS (114.37 ± 0.31 mg/g) and SOA (52.36 ± 1.51 mg/g) radical-scavenging activities. The DPPH activity for Yunnan samples was 8- to 11-fold higher than that of plants from other production areas. In addition, Zhejiang had the lowest level of DPPH (24.32 ± 2.55 mg/g) radical-scavenging activity, while Chongqing samples represented the lowest levels of ABTS (41.68 ± 4.73 mg/g) and SOA (8.11 ± 0.06 mg/g) radical-scavenging capacities. Samples from Zhejiang (43.57 ± 0.83 mg/g) and Anhui (44.79 ± 1.05 mg/g) had similar SOA activity. All these results demonstrate that the dried fruits of *C. speciosa* from Yunnan had higher DPPH, ABTS and SOA radical-scavenging capacities than plants from other production areas. In addition, *α*-glucosidase inhibitory activities of *C. speciosa* from four production areas were also investigated and their results varied vigorously from each other. As shown in Figure 3B, the value of *α*-glucosidase inhibitory activity was in the order of Chongqing (56.59 ± 6.50 mg/g) < Zhejiang (86.41 ± 4.57 mg/g) < Anhui (172.62 ± 3.69 mg/g) < Yunnan (207.23 ± 0.26 mg/g). It could be deduced that some environment factors [19], such as sunshine, soil and rainfall in different production areas affect the chemical profiles of *C. speciosa*, which could further affect the antioxidant and *α*-glucosidase inhibitory activities of *C. speciosa*.

### 2.3. Correlation Analysis

The correlations of TF, TP, polysaccharide, OA and UA contents with antioxidant activity and *α*-glucosidase inhibitory activity of *C. speciosa* were examined (Table 5). TF content (*p <* 0.01) was positively correlated with DPPH (*r* = 0.88), ABTS (*r* = 0.94), SOA (*r* = 0.74) and *α*-glucosidase inhibitory (*r* = 0.97) activities. The same was true for TP with DPPH (*r* = 0.93, *p <* 0.01), ABTS (*r* = 0.97, *p <* 0.01), SOA (*r* = 0.66, *p <* 0.05) radical-scavenging activities but not for *α*-glucosidase inhibitory activity. These results demonstrated that TF and TP possess strong DPPH and ABTS radical-scavenging capacities. The correlation coefficient of TF (or TP) with SOA was lower than that with DPPH or ABTS. The phenolic group in TF and TP would react more easily with DPPH or ABTS radicals than with SOA radicals. Polysaccharide content was correlated with ABTS (*r* = 0.59, *p <* 0.05), SOA (*r* = 0.81, *p <* 0.01) radical-scavenging activities and *α*-glucosidase inhibitory activity (*r* = 0.68, *r* < 0.05). The lower correlation coefficients of polysaccharide with ABTS than TF or TP might account for the different reaction rates of alcohol and phenolic groups with ABTS radical. A comparison of the correlation coefficients of polysaccharide and TP (or TF) with SOA suggested that polysaccharide could better inhibit the formation of superoxide radical and exhibit stronger SOA radical-scavenging activity than TP (or TF). Furthermore, in the present study, there was no correlation of OA level with DPPH, ABTS, SOA and *α*-glucosidase inhibitory activities, whereas UA content was correlated with DPPH (*p <* 0.05) and ABTS (*p <* 0.05) activities but not with SOA radical-scavenging and *α*-glucosidase inhibitory activities [13]. All above results indicated that TF, TP, polysaccharide and UA could be the major active constituents for the antioxidant activity of *C. speciosa*, while TF and polysaccharide were the main components for *α*-glucosidase inhibitory activity of the plant.

## 3. Materials and Methods

### 3.1. Plant Materials and Reagents

The fruits of *C. speciosa* were collected during the mature period from Yunnan, Chongqing, Zhejiang and Anhui provinces. The samples were cut in half vertically and dried under sunlight for 10 days. They were then powdered and stored at 4 °C until use.

Folin-Ciocalteu reagent, phenazine methosulfate (PMS), nicotinamide adenine dinucleotide (NADH), Tris buffer, nitrotetrazolium blue chloride (NBT), 4’-nitrophenyl-beta-d-glucopyranoside (PNPG), *α*-glucosidase (750 U, yeast) and standard free amino acids (aspartic acid, glutamic acid, cysteine, serine, glycine, histidine, arginine, threonine, alanine, proline, tyrosine, valine, methionine, isoleucine, leucine, phenylalanine, lysine and tryptophan) were purchased from Sigma-Aldrich (St. Louis, MO, USA). Methanol, ethanol, acetone, acetic acid, sodium nitrite, sodium hydroxide, hydrochloric acid, phenyl isothiocyanate, triethylamine, sodium acetate, d-(+)-glucose, phenol, 1,4-dioxane, phenylisothiocyanate, sulfuric acid and high-performance liquid chromatography-grade acetonitrile (ACN) were from Sinopharm Chemical Reagent Co. (Shanghai, China). Aluminum chloride, potassium persulfate and UA were from Aladdin (Shanghai, China). Gallic acid was from J&K Co. (Beijing, China). DPPH, OA, oxalic acid, oleic acid, malic acid, citric acid, linoleic acid, palmitic acid and stearic acid were from Macklin Biochemical Co. (Shanghai, China). Chloroform, sulfosalicylic acid, hexane and quercetin were obtained from Solarbio (Beijing, China). ABTS was from Tokyo Chemical Industry (Tokyo, Japan). Deionized water was prepared using a Unique-R20 purification system (Xiamen Oshince Technology Co., Xiamen, China).

### 3.2. Analysis of Chemical Composition

#### 3.2.1. Analysis of TF and TP

*C. speciosa* samples (0.1 g) were extracted twice with 80% ethanol solution (*v*/*v*, 10 mL) in an ultrasonic bath at 60 °C for 40 min. The extracts were combined and stored at 4 °C until use.

TF content was measured as previously described [20], with some modifications and with quercetin as the standard. The extraction solution was diluted for 2 times. Then the obtained sample solution (50 μL) and 0.066 mol/L sulfite solution (100 μL) were dispensed into a 96-well plate. After 5 min, 10% AlCl_3_ solution (*w*/*v*, 15 μL) was added and the mixture was incubated at room temperature for 6 min. A 0.5 mol/L NaOH solution (100 μL) was added to terminate the reaction and the absorbance of the mixture at 510 nm was measured with a microplate reader. TF content is expressed as milligrams of quercetin per gram of dry weight of the plant material.

TP content was determined according to the Folin-Ciocalteu method [21], with some modifications. The extraction solution was diluted for 2 times. Then the obtained sample solution (25 μL) and 10% Folin-Ciocalteu/H_2_O (*v*/*v*, 25 μL) solution were mixed in a 96-well plate. After 5 min, H_2_O (100 μL) and 20% Na_2_CO_3_ solution (*w*/*v*, 25 μL) were added. The plate was maintained in the dark for 30 min and the absorbance was measured at 760 nm with a microplate reader. The results are expressed as milligrams of gallic acid equivalent (mg GAE) per gram (g) of the dry weight of the plant material.

#### 3.2.2. Polysaccharide Analysis

The polysaccharide content was determined according to the Entry-Exit Inspection and Quarantine industry standards of China (SN/T 4260-2015) [22]. Powdered *C. speciosa* sample (0.2 g) was extracted twice with 80% ethanol/H_2_O solution (*v*/*v*, 12.5 mL) in an ultrasonic bath at 80 °C for 30 min. The extracts were combined and the mixture was centrifuged at 4000 rpm for 10 min; the supernatant was discarded. The insoluble material was extracted three times with H_2_O (10 mL) by bath-sonication at 120 W and 80 °C for 30 min. The extracts were combined and the mixture was diluted to 100 mL with H_2_O.

Polysaccharide content was analyzed according to the phenol-sulfuric acid method. Sample solution (1 mL), 5% phenol/H_2_O (*w*/*v*, 1 mL) and sulfuric acid (5 mL) were mixed in centrifuge tubes. After 10 min, the centrifuge tubes were incubated in a water bath at 30 °C for 20 min, the reaction solution was removed to 96-well plates and the absorbance was measured at 490 nm with a microplate reader. d-(+)-glucose was used to generate the calibration curve.

#### 3.2.3. Free Amino Acid Analysis

Powdered samples of *C. speciosa* (3 g) were extracted three times with 0.02 mol/L HCl solution (1.5 mL) in an ultrasonic bath at 30 °C for 5 min. The pooled extracts were allowed to stand in the dark for 2 h before centrifugation at 4000 rpm for 2 min. The supernatant (1 mL) was mixed with 7% sulfosalicylic acid (*w*/*v*, 1 mL) solution with shaking for 1 min. The mixture was maintained in the dark for 1 h and centrifuged at 15,000 rpm for 15 min. The supernatant (500 μL) was mixed with 1 mol/L triethylamine/ACN solution (250 μL) and 0.1 mol/L phenylisothiocyanate/ACN solution (250 μL). After 1 h, hexane (2 mL) was added and the mixture was shaken vigorously. The bottom layer was passed through a 0.22-μm filter and stored at 4 °C until use.

Free amino acid contents were determined according to the previous method with some modifications [23]. Samples were analyzed with an ultra-high-performance liquid chromatography (UPLC) 3000 system (Thermo Fisher, Shanghai, China). An analytical column (4.6 × 250 mm stainless steel, 5-μm C18 silica particles) was used for separation. Mobile phases A (0.1 mol/L sodium acetate solution/H_2_O) and B (80% ACN/H_2_O solution, *v*/*v*) were used for gradient elution as follows: 0–14 min, 0–15% B; 14–29 min, 15–34% B; 29–30 min, 34–100% B; 30–37.1 min, 100%–0 B; 37.1–45 min, 0% B. The flow rate was 1.0 mL/min and the temperature was maintained at 40 °C. The absorbance was measured at 254 nm.

#### 3.2.4. Organic Acid Analysis

Organic acid content was estimated based on Liu’s method [18] with some modifications. Powdered *C. speciosa* (0.5 g) sample was extracted twice with 7% sulfuric/methanol (*v*/*v*, 10 mL) solution at 0 °C for 30 min. The extracts were combined and the mixture was immersed in a water bath at 60 °C for 24 h and then centrifuged at 3000 rpm. The supernatant was sequentially combined with H_2_O (20 mL) and chloroform (20 mL). After 2 h, sodium sulfate (3 g) was added to the sample solution and the bottom layer was used for analysis.

GC-MS analysis of organic acids was performed with a 5977A mass spectrometer system (Agilent Technologies, Santa Clara, CA, USA) and an HP-5ms ultra-inert column (30 m × 250 μm × 0.25 μm). Helium was used as the carrier gas at a flow rate of 1 mL/min. The temperature program was as follows: the column was held at 220 °C for 1 min before increasing the temperature to 228.5 °C at a rate of 5 °C/min and holding for 5 min. The column temperature was increased to 240 °C at a rate of 5 °C/min and held for 1 min, then increased to 290 °C at a rate of 10 °C/min and held for 3 min. The injection volume was 1 μL.

#### 3.2.5. Analysis of OA and UA Contents

OA and UA were extracted using the same method as TP and TF. OA and UA contents were determined with a UPLC system (Waters, Shanghai, China) equipped with an ultraviolet detector. The analysis was performed according to the Pharmacopoeia of the People’s Republic of China [24], with some modifications. A reversed-phase C18 column (2.1 × 100 mm, 1.7-μm C18 silica particles) was used for separation at a temperature of 35 °C. The mobile phase consisted of methanol, H_2_O, acetic acid and triethylamine at a volume ratio of 265:35:0.1:0.05. The flow rate and injection volume were 0.3 mL/min and 5 μL, respectively. Absorbance was measured at 210 nm with a microplate reader.

### 3.3. Antioxidant Activity

The extraction method for assessing the antioxidant activity of *C. speciosa* was the same as that used for TF and TP extractions. The DPPH radical-scavenging activity was evaluated based on a previous report [25]. The extraction solution was diluted for 10 times. Then the obtained sample solution (50 μL) was mixed with 0.12 mg/mL DPPH/ethanol solution (150 μL) and the reaction was incubated at 37 °C for 30 min in the dark. The absorbance was measured at 517 nm and methanol was used as the positive control. The DPPH radical-scavenging rate was calculated according to the formula *A =* (*A_0_* − *A_i_*)/*A_0_* × 100, where *A*, *A_0_* and *A_i_* represent the scavenging rate and absorbance of blank and sample, respectively.

ABTS free radical-scavenging capacity was determined as previously described [26]. Equal volumes of 1.1 mg/mL ABTS/ethanol (50 μL) and 0.68 mg/mL potassium persulfate aqueous (50 μL) solutions were mixed and allowed to stand overnight in the dark. The resultant ABTS free radical working solution (150 μL) and sample solution (50 μL) were added to a 96-well plate and the reaction was allowed to proceed at room temperature for 30 min. The absorbance of the mixture was measured at 734 nm.

The SOA radical-scavenging effect was determined according to a published method [27]. Briefly, 0.3 mmol/L NBT (50 μL) and 0.936 mmol/L NADH (50 μL) were mixed with the sample solution (50 μL) by shaking for 2 min. The mixture was combined with 0.12 mmol/L PMS (50 μL) solution and then maintained at 26 °C for 5 min. The absorbance was determined at 560 nm.

DPPH, ABTS and SOA radical-scavenging activities are all evaluated with a microplate reader and expressed as milligrams of quercetin equivalents per gram dry weight of the plant material.

### 3.4. α-Glucosidase Inhibitory Activity

The extraction method for evaluating *α*-glucosidase inhibitory activity of *C. speciosa* was the same as that used for TF and TP extractions. The *α*-glucosidase inhibitory activity was determined according to Hattori’s method [28] with some modifications. The extraction solution was diluted for 20 times. Then the obtained sample solution (20 μL) and 2.0 mmol/L PNPG (20 μL) were added into the phosphate buffer (50 μL, pH 6.8) in sequence and the mixture was put at 37 °C for 10 min. After addition of 0.9 U/mL enzyme (10 μL), the mixture was further put at 37 °C for 20 min. Finally, 1.0 mol/L Na_2_CO_3_ (150 μL) was added to end the reaction. The absorbance was determined at 405 nm with a microplate reader. *α-*Glucosidase inhibitory activity is expressed as milligrams of quercetin equivalents per gram dry weight of the plant material.

### 3.5. Statistical Analysis

Data are expressed as mean ± standard error of mean of triplicate measurements. Statistical analysis was performed using SAS v.9.2 software (SAS Institute, Cary, NC, USA). Significant differences were determined at *p* < 0.05 by analysis of variance analysis (Duncan’s multiple range test) and the least significant difference method.

## 4. Conclusions

In this study, we compared the chemical composition, antioxidant activity and *α*-glucosidase inhibitory activity of the dried fruits of *C. speciosa* from four production areas (Yunnan, Chongqing, Zhejiang and Anhui provinces) and examined the relationship of the chemical composition of the plant with antioxidant activity and *α*-glucosidase inhibitory activity. The results show that *C. speciosa* has rich chemical composition, good antioxidant activity and *α*-glucosidase inhibitory activity and *C. speciosa* from different production areas exhibit variations in chemical composition, possibly due to factors such as sunlight, soil, rainfall and agricultural operations. The abundant light and heat resources in Yunnan might contribute to the high contents of TF, TP and polysaccharides. A correlation analysis indicated that TF, TP, polysaccharides and UA were the major components responsible for the antioxidant activity of *C. speciosa*, while TF and polysaccharide were the main contributors for *α*-glucosidase inhibitory activity of the plant. The results reveal that *C. speciosa* could be a potential source of natural antioxidants and hypoglycemics in the food industry. The study would be helpful for evaluating the quality of *C. speciosa* in the different production areas. 

## Figures and Tables

**Table 1:** Free amino acid content of the dried fruits of *C. speciosa* from four production areas.

Free Amino Acids	Content (mg/kg) in Samples from Each Region			
	Yunnan	Chongqing	Zhejiang	Anhui
Aspartic acid	149.59 ± 17.04 ^b^	126.30 ± 7.43 ^b^	129.90 ± 9.50 ^b^	263.09 ± 34.02 ^a^
Glutamic acid	298.59 ± 22.74 ^a^	98.47 ± 4.70 ^c^	140.77 ± 12.98 ^b^	133.99 ± 23.63 ^b^
Cysteine	38.64 ± 1.28 ^b^	44.87 ± 1.97 ^a^	38.70 ± 0.95 ^b^	37.95 ± 3.59 ^b^
Serine	1115.38 ± 56.82 ^a^	381.12 ± 30.28 ^b^	1036. 40 ± 67.80 ^a^	1144.61 ± 109.20 ^a^
Glycine	45.01 ± 0.68 ^a^	36.45 ± 2.93 ^b^	33.17 ± 1.88 ^b^	28.63 ± 3.12 ^c^
Histidine	65.16 ± 1.55 ^b^	51.10 ± 0.91 ^c^	73.10 ± 0.85 ^a^	46.30 ± 3.54 ^d^
Arginine	729.13 ± 15.20 ^a^	329.85 ± 51.32 ^c^	673.70 ± 25.82 ^c^	600.75 ± 62.15 ^c^
Threonine	26.58 ± 0.88 ^a^	5.94 ± 0.56 ^c^	26.10 ± 4.20 ^a^	20.78 ± 2.32 ^b^
Alanine	183.20 ± 20.71 ^a^	79.75 ± 2.90 ^c^	56.07 ± 1.69 ^c^	118.78 ± 14.19 ^b^
Proline	106.64 ± 25.75 ^c^	278.21 ± 4.99 ^b^	382.63 ± 23.17 ^a^	294.51 ± 60.17 ^b^
Tyrosine	47.35 ± 1.92 ^b^	36.94 ± 3.91 ^c^	59.73 ± 1.61 ^a^	33.26 ± 0.57 ^c^
Valine	51.42 ± 1.97 ^a^	33.97 ± 1.69 ^b^	30.40 ± 0.62 ^c^	31.61 ± 1.55 ^b,c^
Methionine	30.29 ± 4.54 ^b^	30.32 ± 0.78 ^b^	33.63 ± 0.31 ^a,b^	35.79 ± 0.62 ^a^
Isoleucine	13.78 ± 0.75 ^b^	30.43 ± 3.43 ^a^	14.27 ± 0.47 ^b^	16.14 ± 4.45 ^b^
Leucine	9.41 ± 1.49 ^c^	21.25 ± 2.86 ^b^	34.23 ± 1.40 ^a^	19.87 ± 6.61 ^b^
Phenylalanine	107.75 ± 6.63 ^a^	43.60 ± 2.46 ^c^	44.10 ± 1.51 ^c^	55.41 ± 4.22 ^b^
Lysine	25.35 ± 1.67 ^b^	24.09 ± 0.99 ^b,c^	44.07 ± 2.65 ^a^	21.03 ± 1.74 ^c^
Tryptophan	368.33 ± 16.92 ^c^	634.00 ± 13.75 ^a^	375.33 ± 31.02 ^c^	447.33 ± 15.82 ^b^
Total amino acids	3411.60	2286.66	3226.30	3349.83
Essential amino acids	632.91	823.60	602.13	647.96

Results are shown as mean ± SD (*n* = 3). Different letters in each column indicate significant differences (*p* < 0.05).

**Table 2:** Proportion of organic acids in the dried fruits of *C. speciosa* from four production areas.

Organic Acids	Retention Time (min)	Percentage (%) in Samples from Each Region			
		Yunnan	Chongqing	Zhejiang	Anhui
Oxalic acid	4.37	1.08	0.89	2.00	0.39
Propanedioic acid	5.51	0.11	0.13	0.23	0.13
Fumaric acid	6.59	0.07	0.20	0.13	0.14
Succinic acid	6.69	0.26	0.24	0.42	0.24
Benzoic acid	7.43	0.34	0.74	2.31	0.35
Malic acid	7.76	66.77	16.51	40.58	63.06
Dimethyoxy succinate	7.93	0.15	0.27	0.85	0.49
Cinnamic acid	10.26	0.12	0.17	0.38	0.09
Trans-aconitic acid	10.63	0.12	0.13	0.19	0.13
Citric acid	10.93	2.05	4.22	14.86	3.17
Isocitrate	11.27	0.28	0.19	0.41	0.35
Azelaic acid	11.56	0.03	0.09	0.20	0.10
Phthalic acid	14.08	0.06	0.06	0.17	0.20
Palmitic acid	14.48	4.48	9.07	13.65	5.59
Linoleic acid	16.15	6.21	15.95	8.37	4.76
Oleic acid	16.21	10.86	41.77	9.47	14.73
Stearic acid	16.47	0.89	1.70	1.71	0.81
Arachidonic acid	19.10	0.12	0.46	0.15	0.16
10-Hydroxy-hexadecanoic acid	20.41	6.00	7.20	3.92	5.11
Total		100.00	100.00	100.00	100.00

**Table 3:** Organic acid content of the dried fruits of *C. speciosa* from four production areas.

Organic Acids	Retention Time (min)	Content (mg/g) in Samples from Each Region			
		Yunnan	Chongqing	Zhejiang	Anhui
Oxalic acid	4.37	3.20 ± 0.05 ^a^	2.97 ± 0.18 ^a^	2.84 ± 0.07 ^a^	1.33 ± 0.08 ^b^
Malic acid	7.76	124.96 ± 7.43 ^a^	34.81 ± 0.35 ^b^	32.86 ± 0.05 ^b^	139.91 ± 4.24 ^a^
Citric acid	10.93	10.26 ± 0.35 ^d^	22.53 ± 0.03 ^b^	30.91 ± 0.11 ^a^	17.54 ± 0.38 ^c^
Palmitic acid	14.48	5.78 ± 0.28 ^c^	12.04 ± 0.02 ^a^	7.41 ± 0.07 ^b^	7.45 ± 0.13 ^b^
Stearic acid	16.47	1.05 ± 0.004 ^b^	2.23 ± 0.08 ^a^	1.16 ± 0.10 ^b^	1.09 ± 0.01 ^b^

Results are shown as mean ± SD (*n* = 3). Different letters in each column indicate significant differences (*p* < 0.05).

**Table 4:** OA and UA contents in the dried fruits of *C. speciosa* from four production areas.

Triterpenic Acids	Content (μg/g) in Samples from Each Region			
	Yunnan	Chongqing	Zhejiang	Anhui
OA	90.64 ± 7.87 ^c^	79.95 ± 9.21 ^c^	555.98 ± 20.88 ^a^	261.87 ± 19.12 ^b^
UA	339.97 ± 11.86 ^a^	251.56 ± 8.44 ^b^	164.91 ± 6.70 ^c^	321.06 ± 14.64 ^a^
Total	430.61	331.51	720.89	582.93

Results are shown as mean ± SD (*n* = 3). Different letters in each column indicate significant differences (*p* < 0.05). OA, oleanolic acid; UA, ursolic acid.

**Table 5:** Correlations of TF, TP, polysaccharide, OA, UA contents with antioxidant activity and *α*-glucosidase inhibitory activity of *C. speciosa.*

Chemical Component		DPPH	ABTS	SOA	*α*-Glucosidase Inhibitory Activity
TF	*r*	0.88	0.94	0.74	0.97
	*p*	0.0002	<0.0001	0.01	<0.0001
TP	*r*	0.93	0.97	0.66	0.59
	*p*	<0.0001	<0.0001	0.02	0.06
Polysaccharide	*r*	0.51	0.59	0.81	0.68
	*p*	0.09	0.04	0.001	0.02
OA	*R*	−0.50	−0.42	0.35	−0.47
	*p*	0.10	0.18	0.27	0.15
UA	*r*	0.61	0.65	0.27	−0.53
	*p*	0.04	0.02	0.40	0.09

ABTS, 2,2’-azino-bis(3-ethylbenzothiazoline-6-sulfonic acid); DPPH, 2,2-diphenyl-1-picrylhy-drazyl; OA, oleanolic acid; UA, ursolic acid; SOA, super oxygen anion; p, probability; r, correlation coefficient; TF, total flavonoid; TP, total polyphenol.
